# Uncommon Pathogen in an Unexpected Host: A Rare Case of Rothia mucilaginosa Infective Endocarditis in an Immunocompetent Patient Without an Underlying Valvular Disease

**DOI:** 10.7759/cureus.15458

**Published:** 2021-06-05

**Authors:** Ramy Abdelmaseih, Randa Abdelmasih, Mohammed Faluk, Mustajab Hasan

**Affiliations:** 1 Internal Medicine, University of Central Florida College of Medicine, Ocala, USA

**Keywords:** rothia mucilaginosa, infective endocarditis, native valve, mitral valve, immunocompetent host, ivdu

## Abstract

Rothia mucilaginosa is an infrequent opportunistic pathogen that affects immunocompromised patients, with a high affinity to prosthetic devices. Infections in immunocompetent individuals are extremely rare and usually related to pre-existing valvular heart disease. We report the first case of Rothia endocarditis in an immunocompetent patient without an underlying valve disease.

## Introduction

Rothia mucilaginosa, formally known as Stomatococcus mucilaginosus, is a gram-positive coccus that is found as a commensal in the oral cavity and upper respiratory tract. It is an infrequent opportunistic pathogen, mostly affecting immunocompromised patients such as patients with severe neutropenia, HIV infection, malignancy, diabetes mellitus, and liver cirrhosis. Infections in immunocompetent individuals are extremely rare and usually related to pre-existing valvular heart disease, prosthetic valves, and indwelling vascular catheters [1]. Other risk factors for Rothia bacteremia include intravenous drug use (IVDU) [2]. We report the first case, to our knowledge, of Rothia endocarditis in an immunocompetent patient without an underlying valve disease.

## Case presentation

**Figure 1:**
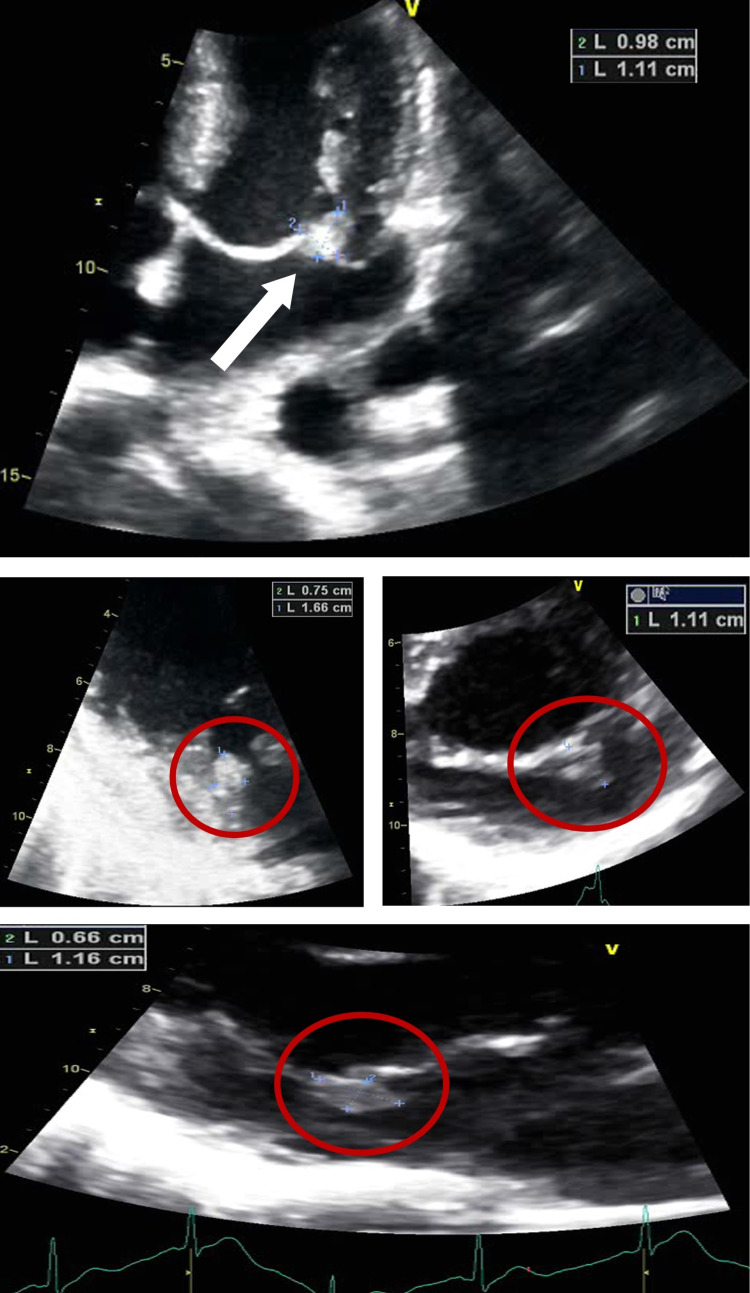
Echocardiographic imaging showing anterior leaflet mitral valve vegetation roughly measuring 1.66 cm x 0.98 cm (white arrow and red circles).

A 46-year-old male with a past medical history significant for remote IVDU and treated hepatitis C virus (HCV), presented with shortness of breath, fatigue, and intermittent low-grade fever for one month. He denied any recent headache, cough, or diarrhea. He also denied any recent IVDU. On presentation, he was febrile at 101 F and had tachycardia at 112 bpm. Cardiovascular examination revealed a holosystolic murmur at the apex. Laboratory workup was remarkable for leukocytosis 35,000 per mm3 with 89% neutrophils. Liver and kidney function tests were normal. HIV screening test was negative. The right upper quadrant ultrasound was negative for cirrhosis. Alpha-fetoprotein was negative. Due to high suspicion of infective endocarditis, the patient was started empirically on intravenous vancomycin. An echocardiogram showed mitral valve vegetation with regurgitation (Figure 1). Blood culture grew Rothia mucilaginosa. The patient was discharged to a subacute care facility with six weeks of intravenous vancomycin.

## Discussion

Rothia mucilaginosa is an emerging opportunistic pathogen associated with prosthetic device infections. Rothia infections include bacteremia, endocarditis, meningitis, pneumonia, bone and joint infection, and cellulitis. Endocarditis is by far the most commonly reported infection [3].

Infective endocarditis is mainly diagnosed using modified Duke criteria that include major criteria (positive blood culture with a typical microorganism consistent with infective endocarditis from two separate blood cultures, and evidence of endocardial involvement) and minor criteria (predisposing risk factors such as IVDU and prosthetic heart valves, fever, vascular phenomena such as septic emboli and Janeway lesions, immunologic phenomena such as Osler nodes and Roth spots, and microbiologic evidence such as positive blood cultures that do not meet the major criteria or serologic evidence of active infection with organism consistent with infective endocarditis). The diagnosis is established in the presence of any of the following: pathologic criteria (pathologic lesions: vegetation or intracardiac abscess demonstrating active endocarditis on histology, or microorganism demonstrated by culture or histology of vegetation or intracardiac abscess) or clinical criteria (two major clinical criteria, one major and three minor clinical criteria, or five minor clinical criteria).

We suspected the possibility of infective endocarditis as the culprit for septicemia in our patient because he met the diagnostic criteria with one major criterion (new valvular regurgitation) and three minor criteria (predisposing factor, fever, and microbiologic evidence).

Upon literature review [4], there were 11 reported cases of Rothia endocarditis, 5/11 were IVDU that had prosthetic valve endocarditis, 3/11 had underlying mitral valve prolapse and developed native valve endocarditis, 2/11 had acute lymphocytic leukemia with neutropenia and developed intraventricular catheter-related ventriculitis and native valve infective endocarditis, respectively, and 1/11 had rheumatic heart disease and developed native valve endocarditis. 

Remarkably, this organism’s prominent adherence properties by producing a biofilm are believed to be a key pathogenic mechanism that increases the risk of catheters and prosthetic cardiac valve colonization in patients with bacteremia. This biofilm is thought to cause disruption of prosthetic valves rendering antibiotics therapy alone ineffective without device removal.

Rothia is generally susceptible to penicillin, ampicillin, cefotaxime, imipenem, and vancomycin. However, partial resistance to penicillin has been reported. Therefore, empirical vancomycin is the drug of choice while awaiting susceptibility testing [5]. A combination of antibiotic therapy and prompt infected device removal is usually necessary for successful treatment. Raising the awareness among physicians of this organism’s potential virulence is needed for better outcomes.

## Conclusions

In conclusion, the presented case was a healthy 46-year-old male, who, despite any known risk factors, developed endocarditis secondary to Rothia mucilaginosa. He was successfully treated with intravenous vancomycin for a total of six weeks. This case highlights the importance of considering Rothia mucilaginosa as one of the causative organisms of endocarditis, even in the most unlikely hosts.
